# Antwerp Civil Hospital

**Published:** 1831-02

**Authors:** 


### ANTWERP CIVIL HOSPITAL.

*A Case of Ununited Fracture of the Thigh-bone, cured by the Application of a Silver Wire between the fractured Extremities.* By Dr. Somme, m.d., Principal Surgeon and Professor in the Civil Hospital of Antwerp.*

An Indian of Madras, a sailor on board the James Scott, whilst in the port of Antwerp, fell from the height of twenty feet, and fractured his left thigh: he was conveyed to the Civil Hospital on the 17th of February, 1828. He appeared to be about thirty, or thirty-five years of age.

The fracture was oblique, and situated about the middle of the thigh: the usual treatment, with two long lateral, and a short anterior splint, bound on with rollers, was employed. His state of health excited no fears for his perfect restoration, but his restlessness brought about a very different result. Many of his shipmates, patients in the hospital, were continually about his bed, and with them he would sit up eating, and amusing himself, and consequently disturbing the bandages as soon as re-adjusted. This mode of proceeding he continued, in spite of all the signs which could be made to him (for he did not understand our language) as to the impropriety of his conduct, and after ten weeks the fracture was as far from union as on the first day; the lower portion of the bone

* Medico-Chirurgical Transactions, vol. iv. p. 371.

riding upon the inside of the upper for about an inch. Extension by means of bandages, with caution to retain the horizontal posture, was employed for six weeks, but without effect, as he still continued as restless as before. M. Larrey's apparatus, with white of egg and tow, were employed, without anticipation of benefit on my part, for a month, but no improvement ensued, although the patient was more tractable: the broken extremities of the bone lying side by side, were connected only by soft substance, and after five months we lost all hope of procuring any bony union.

The means proposed to remedy this annoying accident are, 1st, friction of the fractured extremities of the bone against each other; 2d, amputation; 3d, the introduction of a seton. Amputation, however, can hardly be considered as a mean of cure.

I employed friction for some days, but this painful operation, in consequence of the lateral position of the bony fragments, did not of course act on the extreme ends of the fractured bone, and I soon gave it up, for fear of exciting dangerous consequences.

The friction recommended by the old surgeons has sometimes succeeded. White is said to have cured an ununited fracture of the femur, in this manner. In the Journal de Medicine de Corvisart, Roux et Boyer, cah. iij. there is a case of ununited fracture of the leg cured in the same way. A bandage being applied around the knee, and fastened to the head of the bed, extension was made by a triple pulley, attached to a second bandage applied round the foot. "Three assistants," says the writer, "commenced the extension, by drawing the loose end of the cord: the great power employed completely elongated the limb, and I then violently rubbed the fractured extremities against each other. A bandage was then applied, over it wooden splints, and these enveloped in another roller extending from the toes to the knee, and, finally pads with larger splints. No untoward accident occurred: on the fortieth day the apparatus was removed, and he left his bed on the sixty-ninth day, perfectly cured." I cannot, however, recommend a method, inefficacious, if the efforts to tear asunder the parts are insufficient, and dangerous, if too much violence be used.

Amputation of the fractured extremities of the bone, is an operation alike painful and difficult, and puts the patient in danger of his life; with this view of the case it ought to be rejected from surgical practice. Prudence and reason are equally opposed to that treatment which, for the purpose of remedying a mere inconvenience would endanger the life of an individual.

White, of whom I have just spoken, was the first person who performed this operation upon a child of nine years old, who was the subject of ununited fracture of the humerus. In another case of non-consolidation of the tibia, the same surgeon "made a longitudinal incision, about four inches in length, through the integuments which covered the fracture. By the application of a trephine, he cut off the upper end of the bone; and as the lower end could not be easily sawn off, he contented himself with scraping it. In the course of the subsequent treatment, he had occasion to take off, with the cutting pincers, a small angle of the tibia, and to touch the lower part of the bone with the butter of antimony, as well as to introduce the same caustic between the extremities of the fracture, in order to destroy a substance which intervened. A trifling exfoliation followed. In twelve weeks the bone was firmly united."

One would certainly never attempt to follow so barbarous a practice, more especially in fracture of the leg; since experience proves, that after some time perfect union will take place; of which I could cite many examples, were they not well known to hospital surgeons.

Amputation of the fractured extremities, has been resorted to in ununited fracture of the humerus. M. Larrey mentions a case of this kind under M. Viguerie the younger, the principal surgeon of the hospital at Thoulouse, which was perfectly successful: he does not give any detail of the operation, but justly blames it, as presenting a great number of unsuccessful cases. About fifteen years since, I was present at an operation of this kind, performed by a surgeon at Borgerhout, near Antwerp; it was for an ununited fracture of the arm, which had existed for a long time. The operation was tedious and difficult; the hemorrhage, which was very troublesome, recurred after the operation; a very free suppuration was set up in the space between the ends of the bone, which were kept in their place with great difficulty; and the patient returned home without any union having been effected. In the nineteenth part of the Journal Complementaire du Dictionnaire des Sciences Medicales, there is a case of ununited fracture of the thigh operated on by M. Pezenat, who also cut off the fractured extremities; the patient lived, notwithstanding a very tedious suppuration, followed by exfoliation and sinuses. The cure was not completed till twelve months had elapsed; however, the fracture united.

The third mode of cure proposed is the seton; as it is less dangerous than the other plans, it has been frequently employed. M. Roux, during his visit to England, saw Mr. Bell perform this operation on a child of six years old: he gives the following account of it in his Voyage à Londres, 1814. It was for a fracture of the leg, which had been ununited three years.

"Mr. Bell", says he, "introduced a seton between the fragments of the tibia. For this purpose, he first made a very small incision on the outside of the false joint, then passed the seton between the bony fragments, by means of a strong but slightly curved needle, which was fixed in a handle, and its blade pierced near the point. The skin and other soft parts on the inside of the false joint were pierced with this instrument, armed with fifteen or twenty threads of silk. The result of this operation has not been published."

M. Roux also mentions two other similar operations performed by Mr. Brodie at St. George's Hospital, for false joints of the thigh. The first was in a lad, twelve years old: untoward symptoms occurred: he remained lame, but the fracture was united. The second was a man of thirty, who had also an ununited fracture of the thigh. The silk was removed at the end of nineteen days, on account of the excitement which supervened; a second time the seton was used; it was continued more than five months, but without effecting any union.

Mr. Samuel Cooper, in his Dictionary of Surgery, mentions many other unsuccessful cases. Nor are they surprising, if we recollect that the seton acts only in the course of the silk; and that, suppuration once established, a fistulous ulcer is produced, which leaves the surrounding parts in *statu quo.*

To act upon the fractured extremities, the inflammation must be more extensive. It is recommended to introduce the seton between the fractured extremities, but it frequently happens that one portion riding over the other, they are placed side by side, rather than opposite each other. Such was the case with the individual who was the subject of this paper. I determined therefore to make use of such a plan as would excite inflammation upon a larger surface than the seton could possibly effect, and to keep up the inflammatory action by changing the point of irritation.

The following was my mode of operating:

Let it be remembered, that the left femur was broken obliquely about the middle, and that the fractured extremities rode over each other, the lower inwards, and the upper end outwards.

The patient being placed on his back, and supported, I passed a long trocar and canula at first downwards on the inside of the upper fragment, and made it pierce the skin behind and a little to the outside; the trocar was then withdrawn, and a silver wire passed through the canula and out at the posterior opening. The canula was then withdrawn, and being replaced on the trocar, they were introduced again above and on the outside of the lower fragment, and made to pass out at the same opening behind. The trocar having been removed. the other end of the wire was passed through the canula, so that both ends were in contact behind, leaving a loop in front. I then made an incision in front, from one orifice to the other made by the trocar, and drawing the extremities of the wire through the wound, brought the loop between the fractured ends of the bone, and approximated the edges of the skin with sticking plaster.

In order to secure the tractability of the patient during the treatment, I had a wooden box constructed, sufficiently long to contain the leg and thigh, and so narrow as to serve the purpose of splints. It was lined with pads, and open at top; a hinge corresponding to the bend of the knee allowed the leg to be slightly bent: the outside of the box had also a hinge, so that it could be dropped, and the wounds dressed without moving the limb.

The thigh was enveloped in a six-tailed bandage, the anterior wound brought together with adhesive straps, and the extremities of the wire enveloped in charpie bent outwards.

At each dressing I drew down the wire, so as to depress the loop more and more into the flesh. The anterior wound, which had been made for the introduction of the loop, cicatrized in about fifteen days: the posterior wound afforded but a trifling suppuration. Six weeks after the operation, which was performed on the 12th of August, 1828, the union was distinct, but the wire was not withdrawn till the 2d of October. I then divided one end of the loop near the edge of the wound, and, drawing down the other, removed it completely: the loop had not, however, quite divided the parts which it encircled.

The cicatrization of the wound did not require a very long time, but, in order to ensure the success of the operation, I continued the use of the apparatus for three months after the period at which it was performed. I then allowed him to get up, and walk about on crutches, having applied a pasteboard splint, first soaked in water, to render it capable of taking the form of the limb.

The external projection of the upper extremity of the bone is now nearly absorbed; the two ends are enveloped in a solid mass, which prevents their being felt as before the operation. The patient supports himself on the thigh without any pain, and merely complains of his knee, the motions of which are very limited.

One circumstance which would not have been expected is, that there is no apparent shortening of the fractured limb.

